# Di-μ-chlorido-bis­[chlorido(*N*,*N*′-dibenzyl­propane-1,2-diamine-κ^2^ *N*,*N*′)copper(II)]

**DOI:** 10.1107/S1600536809044997

**Published:** 2009-10-31

**Authors:** Yu-Fen Liu, Da-Fu Rong, Hai-Tao Xia, Da-Qi Wang

**Affiliations:** a Department of Chemical Engineering, Huaihai Institute of Technology, Lianyungang 222005, People’s Republic of China; b Beilun Entry–Exit Inspection and Quarantine Bureau of China, Zhejiang 315800, People’s Republic of China; c College of Chemistry and Chemical Engineering, Liaocheng University, Shandong 252059, People’s Republic of China.

## Abstract

In the title complex, [Cu_2_Cl_4_(C_17_H_22_N_2_)_2_], the Cu^II^ cation is coordinated by a *N*,*N*′-dibenzyl­propane-1,2-diamine ligand and two Cl^−^ anions, and a Cl^−^ anion from an adjacent mol­ecule further bridges to the Cu^II^ cation in the apical position, with a longer Cu—Cl distance of 2.9858 (18) Å, forming a centrosymmetric dimeric complex in which each Cu^II^ cation is in a distorted square-pyramidal geometry. Intra­molecular N—H⋯Cl hydrogen bonding is observed in the dimeric complex.

## Related literature

For Cu—Cl bond distances, see: Alves *et al.* (2004 ▶); Yang *et al.* (2007 ▶).
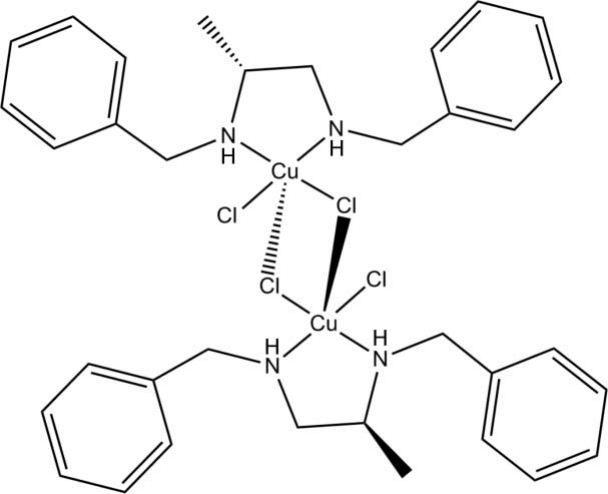


## Experimental

### 

#### Crystal data

[Cu_2_Cl_4_(C_17_H_22_N_2_)_2_]
*M* *_r_* = 777.61
Monoclinic, 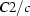

*a* = 21.070 (2) Å
*b* = 13.7377 (17) Å
*c* = 13.2449 (16) Å
β = 114.317 (2)°
*V* = 3493.6 (7) Å^3^
*Z* = 4
Mo *K*α radiation
μ = 1.55 mm^−1^
*T* = 298 K
0.20 × 0.18 × 0.10 mm

#### Data collection

Siemens SMART 1000 CCD area-detector diffractometer
Absorption correction: multi-scan (*SADABS*; Sheldrick, 1996 ▶) *T*_min_ = 0.746, *T*_max_ = 0.860
8528 measured reflections
3077 independent reflections
1858 reflections with *I* > 2σ(*I*)
*R*_int_ = 0.052

#### Refinement

*R*[*F* ^2^ > 2σ(*F* ^2^)] = 0.051
*wR*(*F* ^2^) = 0.104
*S* = 1.06
3077 reflections
199 parameters
H-atom parameters constrained
Δρ_max_ = 0.59 e Å^−3^
Δρ_min_ = −0.67 e Å^−3^

### 

Data collection: *SMART* (Siemens, 1996 ▶); cell refinement: *SAINT* (Siemens, 1996 ▶); data reduction: *SAINT*; program(s) used to solve structure: *SHELXTL* (Sheldrick, 2008 ▶); program(s) used to refine structure: *SHELXTL*; molecular graphics: *SHELXTL*; software used to prepare material for publication: *SHELXTL*.

## Supplementary Material

Crystal structure: contains datablocks I, global. DOI: 10.1107/S1600536809044997/xu2644sup1.cif

Structure factors: contains datablocks I. DOI: 10.1107/S1600536809044997/xu2644Isup2.hkl

Additional supplementary materials:  crystallographic information; 3D view; checkCIF report

## Figures and Tables

**Table 1:** Selected bond lengths (Å)

Cu1—N1	2.034 (4)
Cu1—N2	2.010 (4)
Cu1—Cl1	2.2598 (15)
Cu1—Cl2	2.2663 (17)
Cu1—Cl2^i^	2.9858 (18)

Symmetry code: (i) 
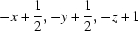
.

**Table 2:** Hydrogen-bond geometry (Å, °)

*D*—H⋯*A*	*D*—H	H⋯*A*	*D*⋯*A*	*D*—H⋯*A*
N2—H2⋯Cl1^i^	0.91	2.51	3.386 (5)	161

Symmetry code: (i) 
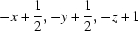
.

**Fig. 1.**
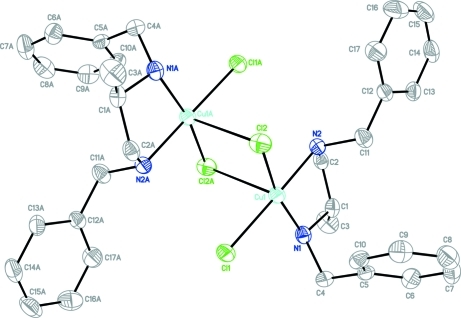
The dimeric complex structure showing the atom-labeling scheme. Displacement ellipsoids are at the 30% probability level. For clarity, H atomes have been omitted [symmetry code: (A) 1/2 - x, 1/2 - y, 1 - z].

## supplementary crystallographic information

### Comment

Copper(II) complexes bridged by a pair of Cl atoms have been widely investigated in both bioinorganic chemistry and coordination chemistry (Yang *et al.*, 2007; Alves *et al.*, 2004). As a further study of the structures of such complexes, the crystal structure of the title complex is reported here.

The molecular structure of the title complex is illustrated in Fig. 1. The Cu^II^ atom are in a distorted square-pyramidal coordination environment (Table 1). The two copper atoms are bridged by a pair of Cl atoms, resulting in complex with centro-symmetric structures. The apical Cu—Cl bond length is 2.9858 (18) Å, whic is longer than 2.737 Å reported by Alves *et al.* (2004), and 2.852 (1) and 2.971 (2) Å reported by Yang *et al.* (2007). The N—H···Cl hydrogen boding is present in the crystal structure (Table 2).

### Experimental

A solution of N,N'-dibenzylpropane-1,2-diamine (1 mmol) in ethanol (20 ml) and a solution of cupric chloride (1 mmol) in ethanol (10 ml) was mixed, the reaction mixture was stirred for 3 h at 323 K. The solution was then cooled slowly to room temperature and filtered. Blue crystals suitable for X-ray diffraction were obtained by evaporation of an ethanol solution.

### Refinement

H atoms were placed in calculated positions with N—H = 0.91 and C—H = 0.93 to 0.97 Å, and refined in riding mode with U_iso_(H) = 1.5U_eq_(C) for methyl H atoms and 1.2U_eq_(C,N) for the others.

### Crystal data

**Table:**

[Cu_2_Cl_4_(C_17_H_22_N_2_)_2_]	*F*(000) = 1608
*M**_r_* = 777.61	*D*_x_ = 1.478 Mg m^−^^3^
Monoclinic, *C*2/*c*	Mo *K*α radiation, λ = 0.71073 Å
Hall symbol: -C 2yc	Cell parameters from 1854 reflections
*a* = 21.070 (2) Å	θ = 2.8–25.3°
*b* = 13.7377 (17) Å	µ = 1.55 mm^−^^1^
*c* = 13.2449 (16) Å	*T* = 298 K
β = 114.317 (2)°	Block, blue
*V* = 3493.6 (7) Å^3^	0.20 × 0.18 × 0.10 mm
*Z* = 4	

### Data collection

**Table:**

Siemens SMART 1000 CCD area-detector diffractometer	3077 independent reflections
Radiation source: fine-focus sealed tube	1858 reflections with *I* > 2σ(*I*)
graphite	*R*_int_ = 0.052
φ and ω scans	θ_max_ = 25.0°, θ_min_ = 1.8°
Absorption correction: multi-scan (*SADABS*; Sheldrick, 1996)	*h* = −25→21
*T*_min_ = 0.746, *T*_max_ = 0.860	*k* = −16→16
8528 measured reflections	*l* = −10→15

### Refinement

**Table:**

Refinement on *F*^2^	Primary atom site location: structure-invariant direct methods
Least-squares matrix: full	Secondary atom site location: difference Fourier map
*R*[*F*^2^ > 2σ(*F*^2^)] = 0.051	Hydrogen site location: inferred from neighbouring sites
*wR*(*F*^2^) = 0.104	H-atom parameters constrained
*S* = 1.06	*w* = 1/[σ^2^(*F*_o_^2^) + (0.0084*P*)^2^ + 19.8791*P*] where *P* = (*F*_o_^2^ + 2*F*_c_^2^)/3
3077 reflections	(Δ/σ)_max_ = 0.011
199 parameters	Δρ_max_ = 0.59 e Å^−^^3^
0 restraints	Δρ_min_ = −0.67 e Å^−^^3^

### Special details

**Table:**

Geometry. All e.s.d.'s (except the e.s.d. in the dihedral angle between two l.s. planes) are estimated using the full covariance matrix. The cell e.s.d.'s are taken into account individually in the estimation of e.s.d.'s in distances, angles and torsion angles; correlations between e.s.d.'s in cell parameters are only used when they are defined by crystal symmetry. An approximate (isotropic) treatment of cell e.s.d.'s is used for estimating e.s.d.'s involving l.s. planes.
Refinement. Refinement of *F*^2^ against ALL reflections. The weighted *R*-factor *wR* and goodness of fit *S* are based on *F*^2^, conventional *R*-factors *R* are based on *F*, with *F* set to zero for negative *F*^2^. The threshold expression of *F*^2^ > σ(*F*^2^) is used only for calculating *R*-factors(gt) *etc*. and is not relevant to the choice of reflections for refinement. *R*-factors based on *F*^2^ are statistically about twice as large as those based on *F*, and *R*- factors based on ALL data will be even larger.

### Fractional atomic coordinates and isotropic or equivalent isotropic displacement parameters (Å^2^)

**Table:**

	*x*	*y*	*z*	*U*_iso_*/*U*_eq_	
Cu1	0.19407 (3)	0.17462 (5)	0.40049 (6)	0.0413 (2)	
Cl1	0.10161 (7)	0.26002 (11)	0.39737 (13)	0.0528 (4)	
Cl2	0.23408 (8)	0.13479 (12)	0.58229 (13)	0.0534 (4)	
N1	0.1603 (2)	0.1813 (3)	0.2330 (4)	0.0419 (11)	
H1	0.1558	0.2458	0.2160	0.050*	
N2	0.2780 (2)	0.1094 (3)	0.3951 (4)	0.0414 (12)	
H2	0.3158	0.1307	0.4549	0.050*	
C1	0.2153 (3)	0.1442 (4)	0.1974 (5)	0.0459 (15)	
H1A	0.2050	0.0758	0.1758	0.055*	
C2	0.2841 (3)	0.1484 (4)	0.2955 (5)	0.0437 (15)	
H2A	0.3182	0.1108	0.2804	0.052*	
H2B	0.3001	0.2154	0.3087	0.052*	
C3	0.2159 (4)	0.2000 (5)	0.0982 (5)	0.071 (2)	
H3A	0.1714	0.1938	0.0368	0.107*	
H3B	0.2514	0.1738	0.0782	0.107*	
H3C	0.2254	0.2675	0.1173	0.107*	
C4	0.0901 (3)	0.1380 (4)	0.1691 (5)	0.0471 (15)	
H4A	0.0782	0.1447	0.0905	0.056*	
H4B	0.0557	0.1736	0.1857	0.056*	
C5	0.0874 (3)	0.0315 (4)	0.1964 (5)	0.0410 (14)	
C6	0.0856 (3)	−0.0402 (5)	0.1225 (5)	0.0537 (17)	
H6	0.0850	−0.0230	0.0542	0.064*	
C7	0.0847 (3)	−0.1367 (5)	0.1488 (7)	0.071 (2)	
H7	0.0830	−0.1846	0.0981	0.085*	
C8	0.0864 (3)	−0.1628 (5)	0.2511 (7)	0.070 (2)	
H8	0.0869	−0.2282	0.2697	0.084*	
C9	0.0873 (3)	−0.0925 (5)	0.3243 (6)	0.0627 (19)	
H9	0.0877	−0.1097	0.3924	0.075*	
C10	0.0877 (3)	0.0050 (5)	0.2968 (5)	0.0471 (15)	
H10	0.0882	0.0528	0.3468	0.057*	
C11	0.2785 (3)	0.0013 (4)	0.4004 (5)	0.0507 (16)	
H11A	0.2485	−0.0240	0.3280	0.061*	
H11B	0.2592	−0.0189	0.4521	0.061*	
C12	0.3497 (3)	−0.0430 (4)	0.4355 (5)	0.0425 (14)	
C13	0.3737 (3)	−0.0720 (4)	0.3563 (5)	0.0493 (16)	
H13	0.3454	−0.0641	0.2813	0.059*	
C14	0.4395 (3)	−0.1126 (4)	0.3890 (7)	0.0607 (19)	
H14	0.4556	−0.1309	0.3361	0.073*	
C15	0.4796 (4)	−0.1254 (5)	0.4962 (8)	0.075 (2)	
H15	0.5233	−0.1537	0.5173	0.090*	
C16	0.4575 (4)	−0.0976 (5)	0.5764 (6)	0.080 (2)	
H16	0.4860	−0.1065	0.6511	0.096*	
C17	0.3918 (3)	−0.0560 (5)	0.5438 (5)	0.0594 (18)	
H17	0.3766	−0.0367	0.5974	0.071*	

### Atomic displacement parameters (Å^2^)

**Table:**

	*U*^11^	*U*^22^	*U*^33^	*U*^12^	*U*^13^	*U*^23^
Cu1	0.0354 (4)	0.0480 (4)	0.0427 (4)	0.0035 (3)	0.0185 (3)	−0.0051 (4)
Cl1	0.0399 (8)	0.0628 (10)	0.0571 (10)	0.0081 (7)	0.0213 (8)	−0.0107 (8)
Cl2	0.0501 (9)	0.0667 (11)	0.0458 (10)	0.0061 (8)	0.0221 (8)	0.0051 (8)
N1	0.046 (3)	0.038 (3)	0.048 (3)	0.001 (2)	0.025 (2)	0.000 (2)
N2	0.039 (3)	0.038 (3)	0.046 (3)	0.002 (2)	0.016 (2)	−0.002 (2)
C1	0.056 (4)	0.039 (4)	0.050 (4)	−0.003 (3)	0.029 (3)	−0.008 (3)
C2	0.043 (3)	0.037 (4)	0.061 (4)	0.000 (3)	0.032 (3)	−0.004 (3)
C3	0.085 (5)	0.083 (5)	0.067 (5)	0.011 (4)	0.052 (4)	0.017 (4)
C4	0.040 (3)	0.052 (4)	0.043 (4)	0.000 (3)	0.010 (3)	−0.003 (3)
C5	0.030 (3)	0.049 (4)	0.038 (4)	−0.001 (3)	0.008 (3)	−0.006 (3)
C6	0.053 (4)	0.060 (5)	0.058 (4)	−0.007 (3)	0.033 (4)	−0.009 (4)
C7	0.068 (5)	0.051 (5)	0.108 (7)	−0.008 (4)	0.052 (5)	−0.023 (4)
C8	0.059 (4)	0.051 (5)	0.107 (7)	0.000 (4)	0.042 (5)	0.009 (5)
C9	0.059 (4)	0.064 (5)	0.065 (5)	−0.004 (4)	0.026 (4)	0.012 (4)
C10	0.039 (3)	0.059 (4)	0.046 (4)	−0.010 (3)	0.020 (3)	−0.009 (3)
C11	0.047 (4)	0.040 (4)	0.070 (5)	0.002 (3)	0.029 (3)	0.004 (3)
C12	0.043 (3)	0.035 (3)	0.051 (4)	0.004 (3)	0.021 (3)	0.002 (3)
C13	0.055 (4)	0.042 (4)	0.053 (4)	0.001 (3)	0.025 (3)	−0.010 (3)
C14	0.064 (5)	0.044 (4)	0.087 (6)	0.004 (3)	0.044 (5)	−0.012 (4)
C15	0.057 (5)	0.060 (5)	0.106 (7)	0.023 (4)	0.032 (5)	0.005 (5)
C16	0.071 (5)	0.089 (6)	0.059 (5)	0.020 (4)	0.006 (4)	0.016 (4)
C17	0.066 (5)	0.069 (5)	0.044 (4)	0.012 (4)	0.024 (4)	0.004 (3)

### Geometric parameters (Å, °)

**Table:**

Cu1—N1	2.034 (4)	C6—C7	1.373 (8)
Cu1—N2	2.010 (4)	C6—H6	0.9300
Cu1—Cl1	2.2598 (15)	C7—C8	1.388 (10)
Cu1—Cl2	2.2663 (17)	C7—H7	0.9300
Cu1—Cl2^i^	2.9858 (18)	C8—C9	1.363 (9)
N1—C4	1.493 (6)	C8—H8	0.9300
N1—C1	1.508 (6)	C9—C10	1.388 (8)
N1—H1	0.9100	C9—H9	0.9300
N2—C2	1.477 (6)	C10—H10	0.9300
N2—C11	1.487 (6)	C11—C12	1.504 (7)
N2—H2	0.9100	C11—H11A	0.9700
C1—C2	1.497 (7)	C11—H11B	0.9700
C1—C3	1.526 (8)	C12—C17	1.352 (8)
C1—H1A	0.9800	C12—C13	1.396 (7)
C2—H2A	0.9700	C13—C14	1.389 (8)
C2—H2B	0.9700	C13—H13	0.9300
C3—H3A	0.9600	C14—C15	1.331 (9)
C3—H3B	0.9600	C14—H14	0.9300
C3—H3C	0.9600	C15—C16	1.378 (10)
C4—C5	1.514 (7)	C15—H15	0.9300
C4—H4A	0.9700	C16—C17	1.393 (8)
C4—H4B	0.9700	C16—H16	0.9300
C5—C10	1.376 (7)	C17—H17	0.9300
C5—C6	1.379 (7)		
			
N2—Cu1—N1	84.19 (18)	C5—C4—H4B	109.2
N2—Cu1—Cl1	174.43 (14)	H4A—C4—H4B	107.9
N1—Cu1—Cl1	92.59 (13)	C10—C5—C6	119.0 (6)
N2—Cu1—Cl2	89.00 (14)	C10—C5—C4	120.1 (5)
N1—Cu1—Cl2	168.46 (14)	C6—C5—C4	120.9 (5)
Cl1—Cu1—Cl2	94.90 (6)	C7—C6—C5	120.6 (6)
N2—Cu1—Cl2^i^	88.13 (13)	C7—C6—H6	119.7
N1—Cu1—Cl2^i^	88.99 (13)	C5—C6—H6	119.7
Cl1—Cu1—Cl2^i^	87.27 (5)	C6—C7—C8	120.0 (7)
Cl2—Cu1—Cl2^i^	100.11 (5)	C6—C7—H7	120.0
C4—N1—C1	113.5 (4)	C8—C7—H7	120.0
C4—N1—Cu1	114.8 (3)	C9—C8—C7	119.9 (7)
C1—N1—Cu1	110.9 (3)	C9—C8—H8	120.0
C4—N1—H1	105.6	C7—C8—H8	120.0
C1—N1—H1	105.6	C8—C9—C10	119.8 (7)
Cu1—N1—H1	105.6	C8—C9—H9	120.1
C2—N2—C11	113.8 (4)	C10—C9—H9	120.1
C2—N2—Cu1	105.7 (3)	C5—C10—C9	120.7 (6)
C11—N2—Cu1	115.6 (3)	C5—C10—H10	119.6
C2—N2—H2	107.1	C9—C10—H10	119.6
C11—N2—H2	107.1	N2—C11—C12	113.9 (4)
Cu1—N2—H2	107.1	N2—C11—H11A	108.8
C2—C1—N1	108.0 (4)	C12—C11—H11A	108.8
C2—C1—C3	112.2 (5)	N2—C11—H11B	108.8
N1—C1—C3	112.3 (5)	C12—C11—H11B	108.8
C2—C1—H1A	108.0	H11A—C11—H11B	107.7
N1—C1—H1A	108.0	C17—C12—C13	118.6 (6)
C3—C1—H1A	108.0	C17—C12—C11	121.0 (6)
N2—C2—C1	110.6 (4)	C13—C12—C11	120.4 (6)
N2—C2—H2A	109.5	C14—C13—C12	120.2 (6)
C1—C2—H2A	109.5	C14—C13—H13	119.9
N2—C2—H2B	109.5	C12—C13—H13	119.9
C1—C2—H2B	109.5	C15—C14—C13	120.0 (7)
H2A—C2—H2B	108.1	C15—C14—H14	120.0
C1—C3—H3A	109.5	C13—C14—H14	120.0
C1—C3—H3B	109.5	C14—C15—C16	121.2 (7)
H3A—C3—H3B	109.5	C14—C15—H15	119.4
C1—C3—H3C	109.5	C16—C15—H15	119.4
H3A—C3—H3C	109.5	C15—C16—C17	119.0 (7)
H3B—C3—H3C	109.5	C15—C16—H16	120.5
N1—C4—C5	112.0 (4)	C17—C16—H16	120.5
N1—C4—H4A	109.2	C12—C17—C16	121.1 (6)
C5—C4—H4A	109.2	C12—C17—H17	119.5
N1—C4—H4B	109.2	C16—C17—H17	119.5
			
N2—Cu1—N1—C4	125.7 (4)	N1—C4—C5—C10	72.6 (6)
Cl1—Cu1—N1—C4	−58.9 (3)	N1—C4—C5—C6	−106.4 (6)
Cl2—Cu1—N1—C4	71.6 (8)	C10—C5—C6—C7	−0.5 (9)
Cl2^i^—Cu1—N1—C4	−146.1 (3)	C4—C5—C6—C7	178.5 (5)
N2—Cu1—N1—C1	−4.6 (3)	C5—C6—C7—C8	−0.8 (10)
Cl1—Cu1—N1—C1	170.8 (3)	C6—C7—C8—C9	1.5 (11)
Cl2—Cu1—N1—C1	−58.7 (8)	C7—C8—C9—C10	−1.1 (10)
Cl2^i^—Cu1—N1—C1	83.6 (3)	C6—C5—C10—C9	0.9 (9)
N1—Cu1—N2—C2	28.3 (3)	C4—C5—C10—C9	−178.1 (5)
Cl2—Cu1—N2—C2	−161.0 (3)	C8—C9—C10—C5	−0.2 (9)
Cl2^i^—Cu1—N2—C2	−60.8 (3)	C2—N2—C11—C12	77.4 (6)
Cl2—Cu1—N2—C11	72.2 (4)	Cu1—N2—C11—C12	−160.0 (4)
Cl2^i^—Cu1—N2—C11	172.3 (4)	N2—C11—C12—C17	87.2 (7)
C4—N1—C1—C2	−151.2 (4)	N2—C11—C12—C13	−93.1 (7)
Cu1—N1—C1—C2	−20.1 (5)	C17—C12—C13—C14	−0.4 (9)
C4—N1—C1—C3	84.5 (6)	C11—C12—C13—C14	179.9 (5)
Cu1—N1—C1—C3	−144.4 (4)	C12—C13—C14—C15	1.0 (9)
C11—N2—C2—C1	79.6 (5)	C13—C14—C15—C16	−1.0 (11)
Cu1—N2—C2—C1	−48.4 (5)	C14—C15—C16—C17	0.4 (12)
N1—C1—C2—N2	45.4 (6)	C13—C12—C17—C16	−0.2 (9)
C3—C1—C2—N2	169.7 (5)	C11—C12—C17—C16	179.5 (6)
C1—N1—C4—C5	69.9 (6)	C15—C16—C17—C12	0.2 (11)
Cu1—N1—C4—C5	−59.2 (5)		

Symmetry codes: (i) −*x*+1/2, −*y*+1/2, −*z*+1.

### Hydrogen-bond geometry (Å, °)

**Table:**

*D*—H···*A*	*D*—H	H···*A*	*D*···*A*	*D*—H···*A*
N2—H2···Cl1^i^	0.91	2.51	3.386 (5)	161

Symmetry codes: (i) −*x*+1/2, −*y*+1/2, −*z*+1.
